# Oral and Periodontal Risk Factors of Prosthetic Success for 3-Unit Natural Tooth-Supported Bridges versus Implant-Supported Fixed Dental Prostheses

**DOI:** 10.3390/diagnostics13050852

**Published:** 2023-02-23

**Authors:** Ioana Cristea, Doriana Agop-Forna, Maria-Alexandra Martu, Cristina Dascălu, Claudiu Topoliceanu, Roland Török, Bianca Török, Dimitrios Bardis, Panagiota Moulavasili Bardi, Norina Forna

**Affiliations:** 1 Faculty of Dental Medicine, Grigore T. Popa University of Medicine and Pharmacy Iasi, Universitatii Street 16, 700115 Iasi, Romania; 2 Implant Institute Török, 1712 Tafers, Switzerland; 3 Bardi Clinic, Ippokratous 166, 114 71 Athena, Greece

**Keywords:** fixed partial dentures, tooth-supported bridge, implant bridge, periodontal disease, peri-implantitis, prosthetic success, smoking

## Abstract

The goals of this research are: (1) to compare the survival and prosthetic success of metal-ceramic 3-unit tooth- versus implant-supported fixed dental prostheses; (2) to evaluate the influence of several risk factors on the prosthetic success of tooth- and implant-supported fixed dental prostheses (FPDs). A total of 68 patients with posterior short edentulous spaces (mean age 61.00 ± 1.325 years), were divided into two groups: 3-unit tooth-supported FPDs (40 patients; 52 FPD; mean follow-up 10.27 ± 0.496 years) and 3-unit implant-supported FPDs (28 patients; 32 FPD; mean follow-up 8.656 ± 0.718 years). Pearson-chi tests were used to highlight the risk factors for the prosthetic success of tooth- and implant-supported FPDs and multivariate analysis was used to determine significant risk predictors for the prosthetic success of the tooth-supported FPDs. The survival rates of 3-unit tooth- versus implant-supported FPDs were 100% and 87.5%, respectively, while the prosthetic success was 69.25% and 68.75%, respectively. The prosthetic success of tooth-supported FPDs was significantly higher for patients older than 60 years (83.3%) vs. 40–60 years old (57.1%) (*p* = 0.041). Periodontal disease history decreased the prosthetic success of tooth- versus implant-supported FPDs when compared with the absence of periodontal history (45.5% vs. 86.7%, *p* = 0.001; 33.3% vs. 90%, *p* = 0.002). The prosthetic success of 3-unit tooth- vs. implant-supported FPDs was not significantly influenced by gender, location, smoking, or oral hygiene in our study. In conclusion, similar rates of prosthetic success were recorded for both types of FPDs. In our study, prosthetic success of tooth- versus implant-supported FPDs was not significantly influenced by gender, location, smoking, or oral hygiene; however, history of periodontal disease is a significant negative predictor of success in both groups when compared with patients without periodontal history.

## 1. Introduction

Posterior short edentulous spaces are a common complaint amongst patients, and although this situation might appear easy to manage in daily practice, the prosthodontists must balance the multiple functional, periodontal, masticatory, esthetic, and occlusal demands that such a situation requires [1]. The clinical decision between the natural tooth versus implant-supported fixed partial dentures (FPDs) is based on anatomic, esthetic, and economic factors, as well as the demands of the patient [2]. The implant–prosthetic treatment provides excellent functional and physiognomic results and decreases psychological trauma compared with the conventional treatment options. However, despite the increased demands for implant–prosthetic rehabilitation, a large category of posterior edentate people cannot benefit from the implant-supported fixed partial dentures therapeutic approach due to social-economic reasons or a combination of local, loco-regional, and systemic factors [3,4].

Considering candidates for implant–prosthetic therapy, the dental practitioner must make his therapeutic decision based on clinical and paraclinical investigations. An important tool for decision-making can be the data collected from retrospective and prospective studies regarding the survival rates, the prosthetic success rates as well as the potential factors that could influence the long-term outcome of the tooth- or implant-supported FPDs [1]. The biological complications (dental caries, endodontic pathology, periodontal disease) and technical complications (loss of retention) can reduce the longevity of tooth-supported FPDs [1,5]. In a study including a 10-year follow-up, FPDs supported by 2–4 natural teeth abutments had a loss risk of 2.1% for abutment fracture, 2.6% for dental caries, and 0.7% for periodontitis, whereas the loss risk due to technical complications was 6.4% (retention loss) and 3.2% (material fracture) [6]. Biological complications (peri-implantitis, as defined by the new classification of periodontal and peri-implant diseases and conditions) and technical complications (loss of retention, screw loosening, abutment fractures, ceramic veneering chipping) can also reduce the prosthetic success of the implant-supported FPDs [7,8,9,10,11]. 

Research comparing tooth-supported bridges versus implant-supported regarding the success rate and the potential risk factors is limited in the literature, and the results are inconclusive [12,13,14,15]. Furthermore, the majority of studies analyze these situations separately, thus a direct comparison is an intriguing perspective.

The goals of this research are: (1) to compare the survival and prosthetic success of metal–ceramic 3-unit teeth- versus implant-supported fixed dental prostheses and (2) to evaluate the influence of several factors on the prosthetic success of teeth- or implant-supported FPDs. 

## 2. Materials and Method

### 2.1. Study Design, Inclusion, and Exclusion Criteria

This retrospective cohort study (with prospective 5–15 years recall) was conducted at Clinical Base of Dental Medicine Faculty, University of Medicine and Pharmacy “Grigore T.Popa” Iasi (Romania), and Bardi Clinic (Athens, Greece), between December 2019 and May 2022. All patients were informed regarding the purpose of the study and signed an informed consent to participate in the study.

This study was performed according to the ethical values of the Declaration of Helsinki and received approval from the ethics committee of U.M.F. “Grigore T. Popa” Iasi (Romania) (Nr.19356). All subjects included in the test groups were informed about the objectives of the research and informed consent was obtained. 

The inclusion criteria were age > 18 years; 3-unit tooth- or implant-supported FPDs with centric pontic (metal–ceramic or ceramic); and follow-up > 5 years. 

The exclusion criteria were refusal to sign the patient consent form, decompensated metabolic diseases, non-compliant patients to periodontal maintenance sessions, pregnancy, radio or chemotherapy during follow-up, cantilever design, or resin-bonded bridges. 

The final study group included 68 posterior edentulous patients (mean age: 61.00 ± 1.325 yrs.; gender: 20 males, 48 females) treated by one surgeon (P.M.B.) between 2005 and 2017. Patients have received 3-unit metal–ceramic tooth-supported FPDs (Group A—40 patients) or implant-supported FPDs (Group B—28 patients) with Nobel (Biocare, Switzerland) implants (length 10–13 mm; width 3.5–4.5 mm). The features of the study group (patients, tooth, and implant-supported FPDs) were reviewed by one investigator (IC) (Table 1). 

### 2.2. Definitions

FPD survival was defined as an FPD remaining in situ with or without complications while still functioning [16]. FPD prosthetic success was defined as a surviving FPD without biological and technical complications [16].

The biological complications associated with prosthetic failure of the tooth-supported FPDs (the abutment tooth can no longer be used as a retention device for RPF) are as follows: extensive cervical caries, extensive periapical lesions, and pathology of a periodontal nature (tooth mobility grade II, deep periodontal pockets (>5.5 mm), extensive alveolar bone loss in the middle 1/3 with or without tooth mobility) [17,18]. The technical complications associated with the prosthetic failure of tooth-supported FPDs are as follows: minor (“chipping”—loss of the ceramic material with or without exposure of the metal that allows chair-side rehabilitation) or major (requires rehabilitation in the dental laboratory) fractures of the ceramic veneers, fracture of the metal framework of the prosthetic restoration, abutment tooth fracture, occlusal wear, and poor marginal adaptation [17,18].

While peri-mucositis is a reversible condition with biofilm removal [19] (Heitz-Mayfeld et al., 2018), peri-implantitis and implant loss were considered biological complications in patients with implant-supported FPDs [16]. The assessment of the peri-implant tissues was carried out using the case definitions exposed in the World Workshop Consensus of Classification of Periodontal and Peri-implant Diseases and Conditions 2017 [7,20] and peri-implant diagnosis criteria of Renvert et al. (2018) [21]:

1. At least one implant surface with the presence of signs of peri-implant inflammation (bleeding on probing and/or suppuration on gentle probing);
2. Radiographic evidence of peri-implant bone loss after initial period of healing;
3. Probing depth increasing when compared to previous values;
4. In the absence of previous radiograph, bone loss of at least 3 mm measured from the shoulder of the implant and probing depth of at least 6 mm associated with bleeding on probing.

The mechanical/technical complications of the implant-supported FPDs are as follows: implant fracture, framework fracture, prosthetic restoration loosening, loss of the screw access hole, minor or major fractures of the ceramic veneers, occlusal wear, and poor marginal adaptation [17].

### 2.3. Data Collection

All subjects included in this study were assessed using a clinical exam and radiologic exams (Rx, CBCT), which investigated FPDs, dental pillars, implants, and abutments status, as well as the surrounding hard and soft tissues. Data were introduced into an SPSS file database (SPSS software, version 27, SPSS Inc., Chicago, IL, USA) by one investigator (IC). The following data were collected:

- Demographic parameters: age group (40–60 yr. vs. >60 yr.) and gender;
- Oral hygiene (OHI-S index: patients treated with tooth-supported FPDs; mPI index: patients treated with implant-supported FPDs);
- Biological complications of the pillar teeth and implants (peri-implantitis);
- Mechanical/technical complications of the tooth- or implant-supported FPDs.

### 2.4. Statistical Analysis

Descriptive statistics including frequencies, means, and standard deviations were calculated for demographic characteristics and follow-up. The risk factors were assessed using statistical tests (Chi-squared) and their strength was evaluated by calculating the corresponding OR (Odds Ratio) where OR < 1 signifies protective factors (which decrease the risk of disease) and OR > 1 signifies risk factors (which increase the risk of disease).

All tests of significance were evaluated at the 0.05 error level with SPSS v.27.0 (IBM, Armonk, NY, USA). Variables that showed statistically significant differences in univariate analysis of prosthetic success were introduced into the multivariable logistic regression analysis. 

## 3. Results

Table 2 and Table 3 detail the description of the study groups and their demographic characteristics.

The survival rate of the tooth-supported FPDs was 100%, while the survival rate of the implant-supported FPDs was 87.5% (Table 4). 

Significant statistical differences were found between the group of tooth-supported FPDs and implant-supported FPDs, regarding the rates of survival (*p* = 0.19) (Table 4). 

The prosthetic success was 69.25% for tooth-supported FPDs and 68.75% for implants-supported FPDs (Table 5).

The absence of significant statistical differences was found between the group of tooth-supported FPDs and implant-supported FPDs, regarding prosthetic success (*p* = 0.963) (Table 5).

Univariate analysis of the tooth-supported FPDs found the absence of a statistically significant association between gender, FPD location (maxillary vs. mandible; quadrants), smoking, and oral hygiene. However, the prosthetic success rate of the tooth-supported FPDs was higher for males (81.8%) vs. females (60%), for mandible (71.4%) vs. maxillary (68.4%), for non-smokers (71.4%) vs. smokers (68.4%), and for patients with excellent and good oral hygiene (77.8%) vs. patients with poor oral hygiene (50%). 

The Chi-squared test yielded significant statistical differences regarding the prosthetic success of the tooth-supported FPDs in patients >60 years old (83.3%) compared to patients in the 40–60 year old age group (57.1%) (*p* = 0.041) as well as in patients with no periodontal history (86.7%) compared to patients with a history of periodontal disease (45.5%) (*p* = 0.001) (Table 6).

The univariate analysis found the absence of a statistically significant association between age groups, gender, FPD location (maxillary vs. mandible; quadrants), smoking, oral hygiene), and prosthetic success of the implant-supported FPDs. However, the prosthetic success rate of the implant-supported FPDs was higher for males (75%) vs. females (66.7%), for mandible (80%) vs. maxillary (63.6%), for non-smokers (71.4%) vs. smokers (50%), and for patients with excellent and good oral hygiene (76.9%) vs. patients with poor oral hygiene (33.3%). 

The Chi-squared test yielded significant statistical differences regarding prosthetic success of the tooth-supported FPDs in patients with no periodontal history (90%) compared to patients with a history of periodontal disease (33.3%) (*p* = 0.002) (Table 7).

In the group of patients with implant-supported FPDs, we identified only a single risk factor, and, therefore, a multivariate analysis was not necessary. Multivariate analyses were used only for the group of patients with tooth-supported FPDs.

For tooth-supported FPDs, a binary logistic regression model was constructed to assess the effects of variables identified as being responsible for statistically significant differences in prosthetic failure of the tooth-supported FPDs, where only two of the identified risk factors were identified. The constructed model was statistically significant (*p* = 0.001—Omnibus Test of Model Coefficients) and explained 43.5% of the variation in prosthetic failure (Nagelkerke R2), correctly classifying 80.8% of cases, compared to 69.2% of cases correctly classified initially using only the constant without any of the possible predictors. 

The variables that showed statistically significant differences in the prosthetic success of the tooth-supported FPDs were introduced into the multivariable logistic regression analysis. 

Age group and periodontal history were found to be predictors of prosthetic success. 

Compared with patients aged older than 60 years, those aged 40–60 years had worse prosthetic success (OR 0.093; *p* = 0.010). 

Patients with periodontal history had significantly less prosthetic success (OR 18.546; *p* = 0.001)

The other variables (gender, location, periodontal history, smoking, oral hygiene, and follow-up) did not show statistical significance relative to prosthetic success (Table 8). 

## 4. Discussion

Treatment decisions as well as the selection of pillar teeth and implant site abutments for fixed partial dentures in patients with short span edentulous spaces should be based on scientific evidence provided by scientific dental research. In the decision-making process, the practitioners must consider pillars with questionable prognosis that can reduce the long-term prosthetic success of the tooth-supported FPDs [22]. 

Our research aimed to respond to a challenging issue for dentists discussing benefits and limits of the fixed prosthetic therapeutic approaches for patients with posterior short span edentulous spaces. The literature reports similar survival rates between 3-unit tooth- and implant-supported FPDs at 1-year follow-up [23] and lower survival and success rates of implant-supported FPDs compared to tooth-supported FPDs for a mean 52-month follow-up [24]. The longevity of the implant-supported FPDs significantly decreased when biological complications and/or mechanical/technical complications occur [25,26,27]. On the other hand, the treatment of the short posterior edentulousness using tooth-supported FPDs involves the preparation of future dental pillars, favoring the accumulation of dental plaque, cervical dental caries, periodontal disease, or periapical pathology [28]. 

Hawthan et al. (2022) reported cumulative survival rates of 90.1% and 77.6% at 5- and 10-years follow-up, respectively, with significantly decreased rates at 67.9% and 52.1% after 15 and 20 years, respectively [18]. A meta-analysis performed by Pjetursson et al. (2007) indicated, for tooth-supported FPDs, survival rates of 93.8% at the 5-year and 89.2% at the 10-year follow-up; for implant-supported FPDs, the survival rates were 94.5% at the 5-year and 89.4% at the 10-year follow-up [26]. High mean follow-up (10.27 ± 0.496 years for tooth-supported FPDs; 8.656 ± 0.718 years for implant-supported FPDs) is an important parameter that contributed to the lower survival rates reported in our study (90.6% for tooth-supported FPDs; 93.6% for implant-supported FPDs). Bart et al. (2012) reported survival rates of 90.4% at 10 years and 80.5% at 15 years for FPDs supported either by natural tooth abutments or implants [28]. Tallarico et al. (2018) reported an 89.2% survival rate for tooth-supported FPDs and an 86.7% survival rate for implant-supported FPDs at the 10-year follow-up [29]. 

In our study, the prosthetic success rates were 63.6% for tooth-supported FPDs and 75% for implant-supported FPDs. These values illustrate a lower success rate than those reported by Pol et al. (2022) at the follow-up (91.7% prosthetic success for 3-unit tooth-supported FPDs; 87.5% prosthetic success for 3-unit implant-supported FPDs) [24]. 

The second part of this research highlighted several factors that have the potential to lead to FPD failures considering the limited availability of data regarding variables complications (demographic parameters, location, periodontal history, smokers, bruxism, materials, and status of the opposing arch) in patients with a short edentulous span. The results of the present study suggest that the prosthetic success of both tooth- and implant-supported FPDs is not significantly influenced by factors such as gender, location, smoking, or oral hygiene. The literature also reports that gender does not influence the prosthetic success of tooth-supported FPDs [17] and implant-supported FPDs [30]. The mandible location increases the failure rate, but without statistical significance, when compared to maxillary tooth-supported FPDs [17]. 

In our study, smoking was not significantly associated with prosthetic failure, but failure rates were higher in smokers when compared to non-smokers both for tooth- and implant-supported FPDs. However, smoking changes the periodontal microbiota, affects the immune system, and increases the incidence and progression of periodontitis, which is associated with a higher risk of tooth loss [31,32]. In addition, smoking significantly decreases the prosthetic success of implant-supported FPDs for patients with a history of generalized aggressive periodontitis [33]. 

Despite the absence of statistical significance, for patients with tooth-supported FPDs, the failure rate was higher when oral hygiene was poor (OHI-S 3.1-6) when compared with patients with excellent or OHI-S 0-3. Poor oral hygiene is an associated risk indicator for implant-supported FPDs [34]. In our study, the age group significantly influenced prosthetic success only in the tooth-supported FPDs. The higher prosthetic success of the tooth-supported FPDs in patients in the age group over 60 years, when compared to the 40–60 year age group (*p* = 0.010), could be explained by better compliance to maintenance sessions and oral hygiene procedures in the former. Patients more compliant to recall and maintenance sessions have better control of dental caries and periodontal disease and thus a lower rate of biological complications and prosthetic failures [35]. 

We also found that patients with a periodontal history had significantly lower rates of prosthetic success both in the tooth- and implant-supported FPDs when compared with patients without a history of periodontal pathology. A systematic review also reported a poorer survival rate and significantly higher risk of peri-implantitis for patients with implant-supported FPDs [36]. The incidence of peri-implantitis and peri-implant marginal bone loss increases significantly in patients with previous tooth loss due to periodontal disease [37,38]. However, implant–prosthetic therapy in periodontitis-susceptible patients is not contraindicated if these patients are compliant with maintenance sessions for the control of bacterial plaque [38]. 

The differences in the prosthetic success rates of the teeth- and implant-supported FPDs from various research groups may be partly attributed to the differences in criteria systems of success and failure as well as different methods of evaluation used across various studies. These issues limit reliable interpretations of data and direct comparisons between study reports [39,40].

Newer therapies in dentistry and implant rehabilitation including ozonated substances, probiotics, paraprobiotics, post biotics, photodynamic disinfection and even other antibiotic substances that are currently not frequently used, could limit the failure rate of bridges, both for natural teeth and implant-supported, thus improving the overall periodontal and oral health of patients [41,42,43,44,45,46].

As limitations of our study, we acknowledge the small number of patients included in the study. Furthermore, we must mention the limitations specific to retrospective studies such as a possible lack of data collected from patients’ files, differences in mean follow-up between study groups, and the absence of radiographic comparisons regarding marginal bone loss around pillar teeth and dental implants. However, retrospective studies, such as this one, assessing potential risk factors for prosthetic success can be more representative of routine clinical practice when compared to prospective studies conducted under highly specific conditions. 

## 5. Conclusions

Similar rates of prosthetic success were recorded for tooth-supported FPDs and implant-supported FPDs. Patients with a history of periodontal disease have significantly lower prosthetic success rates in both tooth- and implant-supported FPDs when compared to patients without a periodontal history.

## Figures and Tables

**Table 1:** Study design (PICO). Components.

Component	Description
Population (P)	Patients with short edentulous span treated either with teeth-supported FPDs or implant-supported FPDs
Intervention (I)	1. Group A: Tooth-supported FPDs 2. Group B: Implant-supported FPDs
Comparison (C)	Inter-groups comparison Group A (Tooth-supported FPDs) vs. Group B (Implant-supported FPDs)
Outcome (O)	Prosthetic success Risk factors (OR)

**Table 2:** Features of the study groups by patients (tooth-supported FPD vs. implant-supported FPD).

	Group A (3-Unit Tooth-Supported FPD)	Group B (3-Unit Implant-Supported FPD)	Total	Pearson Chi-Squared	*p*
Ns (%)	40 (58.9%)	28 (41.1%)	68 (100%)		
Age, m ± SD	57.20 ± 1.597	66.42 ± 1.863	61.00 ± 1.325	-	0.000
Age group, Ns (%)				11.561	0.001
40–60 yr	22 (55%)	4 (14.3%)	26 (38.2%)		
>60 yr	18 (45%)	24 (85.7%)	42 (61.8%)		
Gender, Ns (%)				1.461	0.227
M	14 (35%)	6 (21.4%)	20 (29.4%)		
F	26 (65%)	22 (78.6%)	48 (70.6%)		
Smoking status, Ns (%)				0.622	0.430
Non-smoker	28 (70%)	22 (78.6%)	50 (73.5%)		
Smoker (1–10/day)	12 (30%)	6 (21.4%)	18 (26.5%)		
Periodontal disease History, Ns (%)				0.586	0.444
Yes	18 (45%)	10 (35.7%)	28 (41.1%)		
No	22 (55%)	18 (64.3%)	40 (58.9%)		
Oral hygiene (OHI-S/mPI). Ns (%)				3.631	0.057
0–1	26 (65%)	24 (85.7%)	50 (73.5%)		
2–3	14 (35%)	4 (14.3%)	18 (26.5%)		

**Table 3:** Features of the study groups by FPD (tooth-supported FPD vs. implant-supported FPD).

	Group A (Tooth-Supported FPD)	Group B (Implant-Supported FPD)	Total	Pearson Chi-Squared	*p*
3-units FPD, Ns (%)	52 (61.9%)	32 (38.1%)	84 (100%)		
Follow-up, m ± SD	10.27 ± 0.496	8.656 ± 0.718	9.655 ± 0.417	-	0.060
Follow-up, Ns (%)				7.269	0.007
5–10 yr	20 (38.5%)	22 (68.75%)	42 (50%)		
>10 yr	32 (61.5%)	10 (31.25%)	42 (50%)		
Location (MD/MX), Ns (%)				0.182	0.670
MD	38 (73%)	22 (68.75%)	60 (71.4%)		
MX	14 (27%)	10 (31.25%)	24 (28.6%)		

**Table 4:** Rate of survival (tooth-supported FPDs vs. implant-supported FPDs).

Survival, Ns (%)	Group A (Tooth-Supported FPD)	Group B (Implant-Supported FPD)	Total	Pearson Chi-Pătrat	*p*
YES	52 (100%)	28 (87.5%)	80 (95.24%)	6.825	0.019
NO	0 (0%)	4 (12.5%)	4 (4.76%)		

**Table 5:** Rate of prosthetic success (tooth-supported FPDs vs. implant-supported FPDs).

Prosthetic Success, Ns (%)	Group A (Tooth-Supported FPD)	Group B (Implant-Supported FPD)	Total	Pearson Chi-Squared	*p*
YES	36 (69.25%)	22 (68.75%)	58 (69.05%)	0.002	0.963
NO	16 (30.75%)	10 (31.25%)	26 (30.95%)		

**Table 6:** Tooth-supported FPDs: failure risk factors.

Tooth-Supported FPDs Factors		Prosthetic Success				Total		Pearson Chi-Squared	Failure Risk	
		Yes		No					OR/ RR *	95% CI OR/RR *
		N	%	N	%	N	%			
Location	MD	26	68.4%	12	31.6%	38	100.0%	Chi^2^ = 0.043	-	-
	MX	10	71.4%	4	28.6%	14	100.0%	*p* = 1.000		
Location	1	2	50.0%	2	50.0%	4	100.0%	Chi^2^ = 1.276	-	-
(quadrant)	2	8	80.0%	2	20.0%	10	100.0%	*p* = 0.735		
	3	8	66.7%	4	33.3%	12	100.0%			
	4	18	69.2%	8	30.8%	26	100.0%			
Gender	F	18	60.0%	12	40.0%	30	100.0%	Chi^2^ = 2.836	-	-
	M	18	81.8%	4	18.2%	22	100.0%	*p* = 0.092		
Age	>60	20	83.3%	4	16.7%	24	100.0%	Chi^2^ = 4.161	0.267	0.072 ÷ 0.987
group	40–60	16	57.1%	12	42.9%	28	100.0%	*p* = 0.041 *		
Smoker	Yes	10	71.4%	4	28.6%	14	100.0%	Chi^2^ = 0.043	-	-
(1–10/day)	No	26	68.4%	12	31.6%	38	100.0%	*p* = 1.000		
Periodontal	Yes	10	45.5%	12	54.5%	22	100.0%	Chi^2^ = 10.120	7.800	2.030 ÷ 29.975
history	No	26	86.7%	4	13.3%	30	100.0%	*p* = 0.001 **		
Oral hygiene (OHI-S)	0–3.0	28	77.8%	8	22.2%	36	100.0%	Chi^2^ = 4.012	-	-
	3.1–6.0	8	50.0%	8	50.0%	16	100.0%	*p* = 0.058		
Total		36	69.2%	16	30.8%	52	100.0%			

* Statistically significant, ** Highly Statistically significant.

**Table 7:** Implant-supported FPDs: failure risk factors.

Implant-Supported FPDs Factors		Prosthetic Success				Total		Pearson Chi-Squared	Failure Risk	
		Yes		No					OR/ RR *	95% CI OR/RR *
		N	%	N	%	N	%			
Location	MD	14	63.6%	8	36.4%	22	100.0%	Chi^2^ = 0.857	-	-
	MX	8	80.0%	2	20.0%	10	100.0%	*p* = 0.440		
Location	1	2	50.0%	2	50.0%	4	100.0%	Chi^2^ = 3.762	-	-
(quadrant)	2	6	100.0%		0.0%	6	100.0%	*p* = 0.288		
	3	6	60.0%	4	40.0%	10	100.0%			
	4	8	66.7%	4	33.3%	12	100.0%			
Gender	F	16	66.7%	8	33.3%	24	100.0%	Chi^2^ = 0.194	-	-
	M	6	75.0%	2	25.0%	8	100.0%	*p* = 1.000		
Age group	>60	20	71.4%	8	28.6%	28	100.0%	Chi^2^ = 0.748	-	-
	40–60	2	50.0%	2	50.0%	4	100.0%	*p* = 0.572		
Smoking	Yes	2	50.0%	2	50.0%	4	100.0%	Chi^2^ = 0.748	-	-
	No	20	71.4%	8	28.6%	28	100.0%	*p* = 0.572		
Periodontal	Yes	4	33.3%	8	66.7%	12	100.0%	Chi^2^ = 11.210	18.000	2.717 ÷ 119.23
History	No	18	90.0%	2	10.0%	20	100.0%	*p* = 0.002 **		
Hygiene (mPI)	0–1	20	76.9%	6	23.1%	26	100.0%	Chi^2^ = 4.311		
	2–3	2	33.3%	4	66.7%	6	100.0%	*p* = 0.060		
Total		22	68.8%	10	31.3%	32	100.0%			

* Statistically significant, ** Highly Statistically significant.

**Table 8:** Multivariate analysis regarding significant risk factors of prosthetic success for tooth-supported FPDs.

	Multivariate Analysis—Binomial Logistic Regression			
	Coeff. of Model Equation	*p*-Value	OR	95% CI OR
Age > 60 years	−2.375	0.010	0.093	0.015 ÷ 0.570
Periodontal history	2.920	0.001	18.546	3.120 ÷ 110.239
Constant	−1.346	0.017	0.260	

## Data Availability

The data used to support the findings of this study are available from the corresponding authors upon reasonable request.
